# Edible Substrates for Ready-to-Eat Microgreen Pots: “*Farm on the Fork*” Concept

**DOI:** 10.3390/plants15010049

**Published:** 2025-12-23

**Authors:** Nieves Rodríguez-Sánchez-de-Molina, Victoria Fernández-Lancis, Soundouss Kaabi, Marino B. Arnao, Juan A. Fernández, Catalina Egea-Gilabert, Ginés Benito Martínez-Hernández

**Affiliations:** 1 Food Safety and Refrigeration Engineering Group, Department of Agricultural Engineering, Universidad Politécnica de Cartagena, 30203 Cartagena, Spain; nieves.rodriguez@upct.es (N.R.-S.-d.-M.); victoria.fernandez@edu.upct.es (V.F.-L.); 2 Phytohormones and Plant Development Laboratory, Department of Plant Biology (Plant Physiology), Faculty of Biology, University of Murcia, 30100 Murcia, Spain; soundouss.kaabi@um.es (S.K.); marino@um.es (M.B.A.); 3 Department of Agricultural Engineering, Universidad Politécnica de Cartagena, Paseo Alfonso XIII, 48, 30203 Cartagena, Spain; juan.fernandez@upct.es (J.A.F.); catalina.egea@upct.es (C.E.-G.); 4 Plant Biotechnology Institute, Universidad Politécnica de Cartagena, Campus Muralla del Mar, Edificio I+D+i, 30202 Cartagena, Spain

**Keywords:** mustard, *Brassica juncea*, *Sinapis alba*, flavonoid, gellan gum, edible substrate, flavonoid, phenolic, phytomelatonin

## Abstract

Microgreens are usually grown on non-edible substrates, whereas edible substrates enable a fully edible, ready-to-eat product. This study evaluated mustard (white, red and green) microgreens grown on an edible gellan gum substrate within a “*farm on the fork*” system. Agronomical parameters (dry weight, germination, cotyledon area) and bioactive properties (phenolics, antioxidant capacity, phytomelatonin) were assessed during germination. Essential oil supplementation was also evaluated for sensory and antifungal purposes. Gellan gum at 20 g/L was optimal, supporting high germination (red: 95.7%, green: 98.3%, white: 100% at 72 h) and growth without irrigation. After 9 days, white mustard showed the highest fresh weight (63.1 mg/seedling), hypocotyl length (3.52 cm) and cotyledon area (43.5 mm^2^), while red mustard had the greatest nutraceutical value, with the highest carotenoids (76.4 µg/g FW), flavonoids (4.56 mg/g FW), antioxidant capacity (9.02 µmol TE/g FW) and phytomelatonin (25.5 ng/g FW). Essential oils did not affect biometric traits or antioxidant profile at harvest, although transient rises in flavonoids (+0.34 mg/g FW), antioxidant capacity (+0.97 µmol TE/g FW) and phytomelatonin (two-fold) occurred at early stages (day 3–6). Overall, gellan gum—alone or with essential oils—enabled safe, effective production of ready-to-eat mustard microgreens without compromising growth or nutritional quality.

## 1. Introduction

Microgreens are obtained by germinating seeds in water or simple substrates and are harvested between 7 and 21 days after germination, once the cotyledons are fully expanded and, depending on the species, when the first juvenile true leaf begins to appear. Sprouts, by contrast, are harvested within 4–10 days, before true leaves emerge, and are consumed entirely, including the seed. Baby leaves are harvested when plants reach approximately 8–12 cm in height, typically 30–35 days after sowing. Teen leaves represent an intermediate phase between baby leaves and fully mature plants, showing greater vegetative development but still lacking complete reproductive maturity.

Although originally valued for their aesthetics and concentrated flavors, microgreens have gained particular attention for their high levels of vitamins, minerals, and bioactive phytochemicals—often exceeding those of mature plants [1]—positioning them as promising components within the functional foods sector. Although no specific technical standards exist for microgreens, several regulatory frameworks refer to related products. The Codex Alimentarius provides hygiene guidelines for fresh produce like sprouts; in the EU, microgreens are indirectly covered under Regulations (EU) 208/2013 and (EU) 210/2013 governing sprouts and germinated seeds [2,3]. In the United States, the FDA and USDA classify microgreens as young vegetables distinct from sprouts. In Spain, although no formal definition exists, existing legislation for sprouts—such as the MAPA hygiene guide (2016) and Royal Decree 379/2014—is generally applied [4,5,6].

One of the most extensively studied groups of microgreens is the *Brassicaceae*, including mustard sprouts (*Sinapis alba*), which exhibit particularly high levels of glucosinolates, polyphenols and carotenoids during their early stages of growth [7]. Mustard varieties commonly used for seedling and microgreen production include red mustard (*Brassica juncea* var. *rugosa* ‘Red Frills’), green mustard (*B. juncea* var. *rugosa * ‘Wasabina’) and white mustard (*S. alba*). These species are rich sources of bioactive compounds such as phenolics (e.g., ferulic, sinapic, and caffeic acids, quercetin, kaempferol), phytomelatonin, glucosinolates (particularly aliphatic forms such as sinalbin), and carotenoids (e.g., β-carotene, lutein, zeaxanthin, violaxanthin), among others [7,8,9]. In general, microgreens undergo an active accumulation of these bioactive constituents throughout their short developmental cycle, with concentrations typically peaking near harvest [10].

Conventional substrates used for microgreens include peat, coconut fiber, cellulosic paper, vermiculite, and several biodegradable biotextiles. The substrate plays a fundamental role as a physical support (root anchorage) for germination, subsequent plant growth, and the retention of the water required for plant development. The choice of substrate is critical, as it directly impacts crop productivity and quality [11,12]. These substrates offer good aeration and water-retention properties; however, because they are non-edible, they must be removed during post-harvest handling. This process requires additional manipulation, generates waste, and results in the loss of part of the plant biomass that is not collected (roots and part of the stem) [13,14].

The use of edible substrates for seedling production could significantly reduce waste management requirements while introducing a new concept aligned with the hereby developed “*farm on the fork*” approach. To ensure food safety, such edible substrates must be compatible with thermal treatments that allow proper sterilization, enabling aseptic seedling growth within the final package immediately after sowing. In addition, such substrates must also allow adequate physical support (root anchorage) for germination and subsequent seedling growth. Moreover, the packaging must provide sufficient aeration to prevent oxygen depletion and maintain adequate humidity levels, as seedlings of ≤12 days generally do not require irrigation during their early developmental stages. In addition, the packaging materials employed should be biodegradable and of bio-based origin, supporting a fully circular-economy model within this edible-substrate-packaging system.

Among potential edible substrates that would be sensorially accepted by the consumer, we can propose agar-agar, carrageenan, gelatin, alginates and gellan gum. Agar-agar and carrageenans are polysaccharides derived from red algae and commonly used as gelling agents in food and biotechnological applications. Agar-agar, extracted mainly from *Gelidium* and *Gracilaria* species, forms stable and transparent gels suitable for vegetarian, vegan, halal, and kosher diets, and its high insoluble-fiber content may offer certain physiological benefits [15]. Agar-agar exhibits a characteristic algal flavor at the concentrations required to obtain structurally robust gels, which limits its suitability as neutral, edible substrate for seedling or microgreen production. Gelatin is an animal-derived hydrolyzed collagen that forms thermo-reversible gels but lacks thermal stability above ~80 °C, making it unsuitable for sterilization and unstable at warm temperatures. Its animal origin also excludes vegetarian, vegan, and some religious diets unless certified. Although enzymatic treatments have been proposed to improve its thermal resistance [16], these require complete enzyme inactivation, increase production costs, and reduce environmental sustainability, further limiting its suitability. Gellan gum is a high-molecular-weight anionic polysaccharide produced by microbial fermentation with *Shingomonas elodea*. It is widely used in the food industry as a gelling, stabilizing and thickening agent. Gellan gum forms firm, transparent gels at low concentrations (1.5–2.5 g/L) and remains stable across a broad pH range (3.5–8.0) and at high temperatures (90–120 °C), making it a technically robust material. It is approved as a food additive (E-418) and classified as GRAS by the FDA. Its ability to form thermally stable gels with adjustable textures—depending on acyl content and ionic conditions—distinguishes it from agar and gelatin. Additionally, its microbial origin makes it suitable for vegan, halal, and kosher diets, and it is not associated with allergenicity [17].

Different types of biostimulants have been investigated in seedling production to enhance the accumulation of bioactive compounds. In particular, methyl jasmonate, jasmonic acid, salicylic acid, various salts (e.g., KCl), oligosaccharides, glucose, sucrose, and amino acids (e.g., methionine) have been studied in mustard and other *Brassicaceae* seedlings [7,18,19]. Plant essential oils (EOs) have gained attention due to their strong antimicrobial, antioxidant, antifungal and repellent activities [20]. Beyond these properties, certain EOs may act as biostimulants by modulating plant hormonal regulation, influencing processes such as ripening, stomatal closure and the synthesis of antioxidant metabolites. Hence, daily irrigation with tea tree EO-supplemented solutions has also been reported to increase bioactive compound levels and reduce microbial growth in lettuce seedlings (7 days) [21]. Apart from that study, the application of other EOs as biostimulants has not been studied in seedlings.

The present study aimed to evaluate whether an edible substrate could support the proper development of microgreens from three mustard types—red, green and white mustard—within the newly proposed “*farm on the fork*” concept, which is based on an edible-substrate-packaging system. Specifically, biometric growth parameters (germination rate, fresh weight, hypocotyl length, cotyledon surface area and dry matter) were monitored over a 9-day growth period (22 °C; 16:8 h light:dark). In addition, the contents of key bioactive compounds (total phenolics, flavonoids, phytomelatonin and total antioxidant capacity) were quantified. In a second experiment, the effect of supplementing the edible substrates with EOs was assessed using red mustard microgreens as a model, selected based on the results of the previous experiment without EOs.

## 2. Results and Discussion

### 2.1. Optimization and Selection of the Edible Substrate and Packaging System

Gellan gum is a substrate compatible with sterilisation treatments. In accordance, all studied gellan gum concentrations (13, 18, 20, 23 and 25 g/L) were able to gel after the sterilisation treatment. The concentrations ≥ 23 g/L showed a high-firmness texture that did not allow a deep rooting (only superficial), leading to unsuccessful microgreens development. Conversely, concentrations ≤ 18 g/L did not allow a stable gel to form, leading to gel disintegration when it was demoulded. Similarly, agar-agar concentration of 3 g/L led to an unstable gel, while 14 g/L did not allow proper microgreens rooting. Regarding gelatin, its enzymatic treatment did not allow for obtaining a stable gel after the sterilization process, even at the highest gelatin concentration (60 g/L), transglutaminase concentration (10 g/L) and reaction time (80 min). The unsuccessful enzymatic gelatin treatment in our experiment, compared to that of Calvarro et al. [16], may be due to the different types of gelatin and transglutaminase used in our study compared to the previous one. However, the enzymatic conversion of gelatin is a longer procedure and entails higher economic (enzyme) and energetic (enzymatic incubation) costs, in addition to leaving residual enzymes in the food matrix, which could raise concerns regarding the presence of residual enzyme proteins after ingestion. Finally, agar-agar formed a stable gel at the intermediate concentration (10 g/L) with similar findings compared to gellan gum. Nevertheless, such high concentrations were sensorially rejected (informal sensory tests) due to a characteristic seaweed-like flavour.

Gellan gum at a concentration of 20 g/L was selected as the most suitable substrate in our study. Evensen et al. [22] developed dehydrated, ice-templated, and post-crosslinked alginate–gellan gum scaffolds specifically designed for the microgravity cultivation of *B. juncea* microgreens over 12 days, also without irrigation requirements. Their sophisticated system is necessary in space environments, where the absence of gravitational drainage and convective oxygen transfer can otherwise lead to pore collapse and oxygen limitation. However, unlike our proposed substrate, that gellan gum-based substrate was not edible. In contrast, under terrestrial conditions, our gellan gum substrate offers a simple, efficient, cost-effective, and environmentally friendly solution.

**Figure 1:**
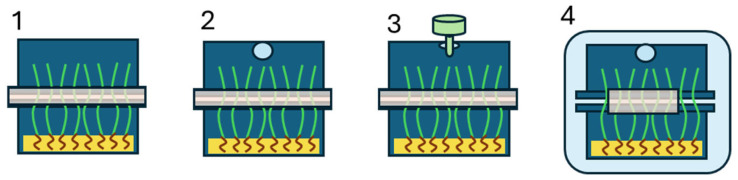
Different packaging systems studied. (**1**) Packaging system 1: Sown tray covered with an empty upside-down tray, edges sealed continuously with adhesive tape; (**2**) packaging system 2: same as system 1, but with a 5-mm hole added to the center of the upper tray; (**3**) packaging system 3: same as system 2, but a 0.22-µm syringe filter inserted into the 5-mm hole; (**4**) packaging system 4: same as system 2, edges sealed discontinuously, with a 5-mm hole and packaged using a PLA flow-pack.

The packaging systems studied (Figure 1) showed different effects. Systems 1 and 2 showed high water condensation levels in the inner side of the packages, which potentially would cause the consumer rejection of this edible-substrate-packaging system. System 3 showed slight condensation, while in system 4, such condensation was not observed. It is also important to note that the use of 0.22 µm membrane filters in our packaging system product would substantially increase its cost. Hence, system 4 was selected based on the previous results and on food-safety considerations, as the flow-pack protects against potential external air contamination. In addition, the biodegradable and bio-based nature of polylactic acid (PLA) used as a flow pack in this system is noteworthy, as it aligns with circular-economy principles.

The selected edible substrate and packaging system was gellan gum dissolved in tap water at 20 g/L at 80 °C and then sterilised at 121 °C for 20 min. The selected packaging system (system 4) consisted of a clamshell-type configuration (two inverted trays, the upper one with a hole), additionally enclosed within a PLA flow-pack (Figure 1).

### 2.2. Characterization of Mustard Microgreens Growth with the Optimized Edible Substrate-Packaging System

#### 2.2.1. Biometric Parameters: Germination Rate, Fresh Weight, Hypocotyl Length, Cotyledon Surface Area and Dry Weight

The red, green, and white mustard showed germination rates of 91.7%, 90.0%, and 98.3% after 48 h, with 95.7%, 98.3%, and 100% after 72 h, respectively. These 72 h germination rates exceed those guaranteed by the supplier (>95%). In the case of white mustard, the observed rate was even higher than the 97.3% previously reported for seeds germinated on coconut fiber [7]. This difference may be attributable to the fact that sprouting performance is largely determined by various factors, including light, temperature, substrate and genetic characteristics, among others [19]. Accordingly, since the temperature and photoperiod conditions were identical in our study and the previous one, gellan gum seems to be a more appropriate substrate for enhancing the germination compared to the coconut fiber used by Artés-Hernández et al. [7]. Nevertheless, differences in the genetic characteristics of the seed lots used in both studies could also influence this outcome.

At 9 days after sowing, red mustard microgreens were the smallest microgreens among the three mustard types, with fresh weight, hypocotyl length and cotyledon surface area values of 17.9 mg, 1.70 cm and 11.0 mm^2^, respectively (Table 1). Green mustard showed intermediate levels between red and white mustard. Red and green mustard followed a similar overall growth trend. On the contrary, white mustard showed the fastest growth, reaching the highest fresh weight, hypocotyl length and cotyledon surface area, with values of 63.1 mg, 3.52 cm and 43.5 mm^2^, respectively (Table 1). White mustard also showed a higher water content, as depicted by its lower dry matter content compared with the other mustard types, which may result in more tender microgreens. Most of the leaf expansion in white mustard occurred between days 3 and 6, with a markedly smaller increase from days 6 to 9, whereas hypocotyl elongation progressed more steadily throughout the entire period. This suggests that the larger size of white mustard microgreens is primarily due to their faster-developing cotyledons.

Published data are consistent with the morphological performance observed in this study. Hypocotyl lengths reported for white mustard grown on filter paper (2.31–3.37 cm at day 7; [23]) and for non-primed seedlings (2.43 cm at day 10; [24]) are comparable to, or slightly lower than, our values, confirming the suitability of gellan gum as a growth substrate. Red mustard grown hydroponically showed faster elongation [25], consistent with the enhanced growth typically observed in nutrient-rich systems. In both white and red mustard, the most pronounced cotyledon expansion occurred between days 3 and 6, with slower growth thereafter, a pattern also observed in white mustard grown on coconut fiber [7].

The slight decrease in fresh weight of white mustard from day 6 to day 9 reflects the onset of a late growth plateau, caused by depletion of seed reserves, absence of external nutrients in the inert substrate, water loss, and early cotyledon senescence. Artés-Hernández et al. [7] similarly reported morphological stabilization in late-stage white mustard microgreens.

Dry matter trends (Table 1) indicate a real loss of biomass in addition to moisture variation, consistent with the mobilization and respiration of storage reserves. This behaviour aligns with general seedling physiology: maize seeds show a progressive reduction in dry matter as endosperm reserves are consumed during early germination [26], and soybean cotyledons can lose up to 70% of their initial dry mass during the first 7–10 days [27].

This growth pattern suggests that white mustard may be better adapted to the gellan gum substrate, maintaining active tissue expansion at later stages. Such mustard type-dependent differences are well documented in *Brassicaceae*, where growth rate and shoot elongation are strongly modulated by genotypic traits [28,29]. In contrast, the stagnation observed in red and green mustard agrees with the early growth plateau previously reported for these microgreens [30].

High water retention is typical of *Brassica* microgreens and reflects rapid cell expansion and thin leaf structures [29]. Despite their overall moisture increase, white mustard maintained a lower fresh weight/dry weight ratio than the other cultivars, suggesting a more compact tissue structure. Final moisture values (>80–90%) were comparable to those reported for mustard microgreens at harvest [31], although such high water content may reduce shelf life due to enhanced microbial and physiological deterioration [32,33]. Despite its higher moisture content, white mustard’s lower fresh weight/dry weight ratio can be disadvantageous because it reduces marketable fresh yield, produces a denser and less tender texture, and may concentrate pungent compounds. These traits can limit its visual appeal, culinary quality, and overall commercial performance as a microgreen.

Based on the previous comments, it would be worthwhile to apply biostimulants (such as EOs, as previously reported in wheat seedlings [34]) to enhance the microgreens’ performance in the lowest-growth mustard type: red mustard.

#### 2.2.2. Pigments

The total chlorophyll contents (chlorophyll a + chlorophyll b) of red, white, and green mustard microgreens at day 9 were 565.7, 241.2, and 160.8 mg/kg FW, respectively (Table 2). Chlorophyll a accounted for 66–72% of total chlorophylls, with no high differences among the three mustard types. These proportions are consistent with the chlorophyll a/b ratios typically reported for red, green, and white mustard seedlings [35,36]. The marked differences in total chlorophyll content reflect distinct temporal patterns of pigment accumulation during early development. In red mustard—and, to a lesser extent, in green mustard—chlorophyll biosynthesis was concentrated between days 3 and 6, indicating a rapid transition toward photosynthetically active tissue. Conversely, white mustard exhibited a more gradual and sustained increase in chlorophyll content throughout days 3–9, indicative of slower but more uniform chloroplast development.

Among the mustard types evaluated, red mustard also exhibited the highest total carotenoid concentration at 9 days (76.4 mg/kg FW), corresponding to a 1.4- to 2.9-fold increase compared with green and white mustard microgreens, which did not differ significantly from each other (*p* > 0.05) (Table 2). In mustard microgreens, lutein and β-carotene have been consistently reported as the predominant carotenoids [37], a pattern that aligns with the pigment composition observed in other Brassica microgreens.

Recent insights into light-regulated photomorphogenesis provide a unified mechanistic explanation for these cultivar-dependent differences in both chlorophyll and carotenoid accumulation. Chlorophyll biosynthesis during de-etiolation is coordinated by multiple photoreceptor families—including phytochromes, cryptochromes, phototropins, and UVR8—whose signaling converges on the COP1–SPA/HY5 regulatory module [38]. HY5 stabilization under light promotes the transcription of genes required for chlorophyll synthesis and chloroplast differentiation, accelerating the formation of photosynthetically competent tissue. This mechanistic framework aligns with the faster chlorophyll build-up observed in red mustard compared with green and white mustard, suggesting a more responsive activation of light-driven developmental pathways in this cultivar.

Carotenoid biosynthesis follows a parallel regulatory logic. Carotenoid accumulation is tightly coupled to the etioplast–chloroplast transition and strongly modulated by phytochrome activity, as first demonstrated in germinating white mustard by Frosch and Mohr [39]. Beyond their photosynthetic roles, carotenoids act as essential photoprotective molecules, preventing the photooxidative degradation of nascent chlorophylls and supporting their stable accumulation [40]. Central to this pathway is the light-induced expression of phytoene synthase—the enzyme catalyzing the first committed step of carotenoid biosynthesis—which is upregulated during de-etiolation and controlled by phytochrome signaling [40,41].

#### 2.2.3. Total Flavonoid Content, Total Phenolic Content, Total Antioxidant Capacity and Malonaldehyde

At 9 days after sowing, red mustard microgreens showed the highest total flavonoid content (4.56 mg/kg FW), which was 14% higher than that of white and green mustard microgreens (Table 3). The latter two showed similar (*p* > 0.05) contents among them. Similarly, Bafumo et al. [10] found higher flavonoid contents in red mustard microgreens compared to white mustard microgreens at 12 days after sowing. In particular, those authors reported quercetin-3-glucoside as the predominant flavonoid, which is well known for its antioxidant, anti-inflammatory and cardioprotective properties [42,43]. During microgreens growth, all mustard types showed a similar increasing flavonoid trend during the true microgreens development stage (days 6 to 9). In particular, the total flavonoid content of the three mustard types slightly increased (3.2–4.2%) from day 6 to day 9 (Table 3). This trend is consistent with a developmental upregulation of secondary metabolism as the photosynthetic apparatus matures and oxidative stress defense mechanisms are reinforced [33].

Regarding the total phenolic content, white mustard microgreens showed the highest content at 9 days (1.30 mg/g FW), which was 23% and 120% higher than those of red and green mustard microgreens (Table 3). A similar trend was repeated in the previous days. Contrary to flavonoid contents, a decreasing trend of the total phenolic content during microgreens growth was observed for green and white mustards. This trend may be related to the metabolic shift described by Park et al. [44], who observed a progressive decline in phenolic acids concomitant with increasing anthocyanin accumulation in *B. juncea* microgreens (12 days).

The markedly higher total flavonoid content observed in red mustard microgreens can be explained by their intrinsic accumulation of anthocyanins and quercetin derivatives, as previously described for pigmented mustard microgreens, where anthocyanin concentration increases sharply during early development [44]. Pigmented *Brassica* varieties are known to concentrate larger amounts of flavonoids than their green counterparts [10], which agrees with the significantly higher values found in red mustard. Conversely, the highest total phenolic content recorded in white mustard microgreens is consistent with reports indicating that non-pigmented mustard types accumulate greater amounts of simple phenolic acids and other non-flavonoid phenolics, which contribute strongly to total phenolic content despite their lower flavonoid levels [10]. This explains why white mustard exhibited the greatest total phenolic content across all sampling days, whereas green mustard consistently showed intermediate or lower levels.

The total antioxidant capacity was highest (9.02 µmol TE/g FW) for red mustard microgreens (9 days), while green and white mustards showed 33 and 74% lower contents compared to red mustard (Table 3). It may be explained by the higher contents of phenolic compounds in red mustard, compared with green and white mustards, as described in the previous section. Correlations of the total antioxidant capacity with total flavonoid and total phenolic contents revealed high R^2^ values of 0.84–0.88. For white mustard, an R^2^ correlation of 0.70 was found between its total antioxidant capacity and total flavonoid content. Interestingly, the total antioxidant capacity–total flavonoid content correlation for white mustard was negative (contrary to the rest of the data). The negative correlation observed for the total flavonoid content may be related to their intrinsically low concentrations in non-pigmented mustard types. At such low levels, the measurements are more affected by analytical variability and by matrix effects from co-extracted simple phenolics, which dominate the total phenolic content determined by the Folin–Ciocalteu method. These interferences can lead to an apparent decrease in total flavonoids over time that is not necessarily biologically meaningful.

Malondialdehyde (MDA), an indicator of lipid peroxidation and oxidative stress, showed clear differences among mustard types throughout microgreen development (Table 3). Overall, red mustard exhibited the highest MDA levels, whereas green mustard consistently showed the lowest values at all sampling times (Table 3). Red mustard consistently showed the highest MDA values, while green mustard exhibited the lowest, indicating lower lipid peroxidation in the latter. On day 6, red mustard reached its maximum MDA content (22.60 nmol/g FW), whereas green mustard remained stable at much lower levels (≈10 nmol/g FW). White mustard showed intermediate values, with a notable decrease by day 9. These trends align with the patterns observed for total antioxidant capacity (TAC). Red mustard, which displayed the highest TAC, also showed higher MDA levels, suggesting that its elevated antioxidant activity reflects a compensatory response to greater oxidative stress. Conversely, the low MDA levels in green mustard are consistent with its lower TAC, indicating reduced oxidative pressure and a lesser requirement for antioxidant upregulation.

#### 2.2.4. Phytomelatonin Content

At 9 days after sowing, red mustard microgreens showed the highest phytomelatonin content (25.5 ng/g FW), while green and white mustard microgreens showed similar (*p* > 0.05) levels of 9.2–13.3 (Table 3). Aguilera et al. [45] also found high phytomelatonin contents in other *Brassica* germinates (red cabbage, 857.4 ng/g DW; radish, 536.3 ng/g DW; broccoli, 439.1 ng/g DW), whereas lower contents were found in legume germinates (alfalfa, 133.4 ng/g DW; mung bean, 166.2 ng/g DW; lentil, 217.3 ng/g DW; and onion, 302.3 ng/g DW). Those authors found that the phytomelatonin content increased by 1.1–2.9-fold from non-germinated to germinated seeds. Saleh et al. [46] found that microgreens cotyledons exhibited higher phytomelatonin levels than the radicles, likely due to their role as the primary metabolic reserve and their greater metabolic and oxidative activity during early seedling development, which enhances antioxidant production, including phytomelatonin. Apart from these studies, the literature on phytomelatonin contents in germinated seeds during microgreen development is very scarce and limited to a few legume germinates (after 6 days of germination), with values of 14–208 ng/g FW (from higher to lower contents: chickpea > common bean > lupine > fenugreek > lentil > broad bean) [47].

During mustard microgreen growth, green and red mustards showed the highest phytomelatonin contents of 28.3 and 25.5 ng/g FW after 6–9 days. The highest phytomelatonin levels in white mustard were observed on day 6. The greatest contents of phytomelatonin in legume germinates have also been reported on day 6 of germination [46], followed by a decrease [47]. These reductions can be explained by phytomelatonin metabolism [45]. Nevertheless, the slower microgreen growth observed in red mustard (see Section 2.2.1) is consistent with a more delayed increase in phytomelatonin for this variety, which reached its highest levels at day 9 (Table 3).

Non-germinated green, red, and white mustard seeds showed phytomelatonin contents of 495.2, 452.9, and 202.8 ng/g DW, respectively. Similar phytomelatonin contents have previously been reported for white mustard seeds [48]. The highest phytomelatonin contents reported in the literature correspond to thyme (38,000 ng/g DW) [49], followed by other plants (peppermint < *Hypericum perforatum* L. < sage < Chinese liquorice), with values ranging from 19,000 to 34,000 ng/g DW, while coffee beans also contain high levels (5800–6800 ng/g DW) (compiled by Arnao & Hernández-Ruiz [48]). However, to the best of our knowledge, the phytomelatonin contents of red (*B. juncea* var. rugosa ‘Red Frills’) and green mustard (*B. juncea* var. rugosa ‘Wasabina’) seeds have not been previously reported.

In general, elevated phytomelatonin concentrations in plants are associated with exposure to natural or experimentally induced stressors, including physical factors (UV radiation, extreme temperatures, high pressure) and chemical challenges (salinity, alkalinity, drought, heavy metals, herbicides, or reactive oxygen species) (reviewed by Arnao & Hernández-Ruiz [48]). Hence, the relatively low phytomelatonin content of mustard microgreens could potentially be enhanced during growth through the application of biostimulants such as essential oils, although the use of these plant extracts as stress-inducing agents to increase phytomelatonin levels has not yet been investigated.

### 2.3. Effect of the Essential Oil Supplementation of the Edible Substrate on the Mustard Microgreen Growth

#### 2.3.1. Biometric Parameters: Germination Rate, Fresh Weight, Hypocotyl Length, Cotyledon Surface Area and Dry Weight

In general, the supplementation of the selected edible substrate (gellan gum at 20 g/L) with EOs did not affect (*p* > 0.05) the studied biometric parameters. Microgreens of the red mustard (at 9 days after sowing) showed fresh weight, hypocotyl length, cotyledon surface area and dry weight values of 17.9 mg, 1.8 cm, 9.8 mm^2^ and 6.75%, respectively. The germination rate was not affected (*p* > 0.05) by the EOs supplementation. However, a reduction in dry weight of microgreens (9 days) was observed (Table 1). This pattern—unchanged fresh biomass and morphology together with reduced dry matter—is physiologically coherent with a mild metabolic stress or allelopathic action that affects biomass deposition more than hydration.

When comparing our results with the literature, an important contrast emerges. Hence, EOs are well documented to exert phytotoxic or allelopathic effects in various plant systems, often inhibiting germination, root elongation or dry biomass. For example, Moura et al. [50] demonstrated that *Piper marginatum* EO induced strong toxicity in lettuce, pepper, tomato and *Spermacoce verticillata*, causing severe leaf necrosis and reductions in plant dry mass in both detached-leaf and post-emergence assays, especially at ≥0.1% concentrations [50]. Similarly, Singh et al. [51] reported that *Curcuma zedoaria* essential oil inhibited germination, slowed seedling growth and markedly reduced dry biomass in lettuce and tomato seedlings, whose root systems were particularly affected. Many of these effects were associated with oxygenated monoterpenes (e.g., 1,8-cineole, camphor), known to impair mitosis, mitochondrial respiration, photosynthesis and membrane integrity.

Despite this well-documented phytotoxic potential, the effects observed in our microgreen system were substantially milder. Unlike the severe growth suppression, necrosis or strong biomass inhibition reported in seedlings or adult plants in the aforementioned studies, our microgreens preserved their morphology, height, leaf area and fresh biomass even in EO-supplemented treatments. Only dry matter decreased, suggesting that early microgreen stages grown on a hydrated edible hydrogel may be less sensitive to EO-induced damage than germinating seeds or mature tissues in soil. Additionally, the nature of our system—short growth cycle, high water availability from the hydrogel matrix, and limited EO volatilisation due to the closed packaging—may have reduced the intensity of allelochemical exposure compared to the direct application methods used in the cited works.

Therefore, while the literature consistently demonstrates that EOs can decrease dry biomass and impair growth in diverse plant models, our findings show that microgreens can tolerate EO supplementation without significant morphological penalties. The reduction in dry weight observed here is thus consistent with the broader allelopathic effects described in previous studies, but manifests in a milder form that does not compromise commercial morphology or fresh yield.

#### 2.3.2. Bioactive Compounds

At 9 days after sowing, microgreens of red mustard with the EOs-supplemented edible substrate showed total flavonoid, total phenolic and total antioxidant capacity values of 4.34 mg/g FW, 0.89 mg/g FW and 6.32 µmol TE/g FW, respectively (Table 4). The EOs supplementation did not affect (*p* > 0.05) the total antioxidant capacity when compared to non-EOs supplemented samples. Accordingly, total flavonoid and total phenolic contents of microgreens (9 days) did not highly change (<0.2 content units) after the EOs supplementation of the edible substrate. Interestingly, total antioxidant capacity and total flavonoid content were augmented by 0.97 and 0.34 units, respectively, on day 6.

Overall, the incorporation of EOs into the edible substrate did not markedly alter the antioxidant profile of 9-day-old red mustard microgreens, as total flavonoids, total phenolics, and total antioxidant capacity remained broadly comparable to the control. The slight decreases detected can be interpreted as a normal physiological adjustment, in which part of the antioxidant pool may be redirected to manage the bioactive influence of the EOs, rather than reflect a true loss of antioxidant potential. Notably, at an earlier developmental stage (day 6), both antioxidant capacity and flavonoid content increased, underscoring the metabolic flexibility of the microgreens. This adaptive response suggests that other health-relevant metabolites—such as phytomelatonin—may also be modulated in meaningful ways.

The literature on the preharvest application of EOs as biostimulants is very limited. Previous research showed that lettuce seeds placed for 7 days on moist filter paper and subjected to a daily spray of tea tree EO exhibited increased total phenolic content, total flavonoid content and total antioxidant activity [21]. However, the same authors did not observe this elicitation effect when seeds were only exposed to a single soaking treatment with tea tree EO, in the absence of daily EO application during germination. In our case, daily irrigation with EOs is not compatible with the edible-substrate-packaging system. For this reason, our research group is currently investigating efficient strategies for controlled and sustained EOs delivery, such as the use of EO-encapsulated active PLA materials or the incorporation of encapsulated EOs directly into the edible substrate. These approaches aim to avoid the “burst effect” associated with sudden EO release—which is restricted to the early stages of germination—and instead provide low but constant EO doses throughout the entire germination period through controlled release mechanisms.

Consistent with the previous findings, phytomelatonin content was 2-fold elicited at the early germination stage (3 days) due to the EO “burst effect”. Nevertheless, such EO-mediated phytomelatonin increment was not observed in the subsequent germination days, showing similar (*p* > 0.05) contents to non-EO-supplemented edible substrate. Hence, a controlled release of EO, such as through encapsulation, would be crucial to achieve effective phytochemical elicitation of bioactive compounds in microgreens at the commercial stage (≥9 days).

## 3. Materials and Methods

### 3.1. Materials and Cultivation Conditions

White mustard (*Sinapis alba*), red mustard (*Brassica juncea* var. *rugosa* ‘Red Frills’) and green mustard (*B. juncea* var. *rugosa* ‘Wasabina’) seeds were provided by CN Seeds (Cambridgeshire, UK). Before sowing, seeds were sanitized with 10% NaOCl for 10 min, followed by three rinses with distilled water. The sanitized seeds were individually and manually sown by placing each seed on the surface of the edible substrate (optimized as described in Section 2.2) at a sowing density of approximately 8000–10,000 seeds per m^2^. Sown packaged trays (optimized as described in Section 2.3) were cultivated in a controlled-environment growth chamber at 22 ± 2 °C with a 16:8 h light:dark photoperiod up to 9 days. Irrigation was not applied. The illumination system of the chamber consisted of fluorescent lights with white full spectrum (0.31 W/m^2^; Philips 36 W/54–765) with a photon flux density of 9.6 ± 0.8 μmol/m^2^ s. Petri dishes were placed 60 cm from the light source.

Three types of edible substrates were evaluated: agar-agar (SOC Chef, Cataluña, Spain), gelatin (Royal, Madrid, Spain) and gellan gum (SOC Chef, Lleida, Spain). Transglutaminase was provided by Doscadesa (Molina de Segura, Spain).

The essential oils (EOs) of lemon, grapefruit, lemongrass, mint, cinnamon, orange, ginger and oregano were acquired from Esencias Martínez Lozano S.A. (Caravaca de la Cruz, Spain).

All these materials were food-grade.

### 3.2. Optimization and Selection of the Edible Substrate and Packaging System

#### 3.2.1. Optimization and Selection of the Edible Substrate

Different substrate concentrations were evaluated to determine the minimum concentration required to form a firm substrate, while avoiding excessive firmness that could hinder seed root development.

Gellan gum was dissolved in tap water at 13, 18, 20 (recommended by the supplier), 23 and 25 g/L with stirring using a heating magnetic plate at 80 °C and then sterilized at 121 °C for 20 min.

Agar-agar powder was dissolved in distilled water using a magnetic stirrer. The concentrations tested were 3, 10 g/L (manufacturer-recommended), and 14 g/L. The homogenized solutions were sterilized in an autoclave at 121 °C for 20 min, as agar-agar is highly thermally stable.

Gelatin was dissolved in tap water at 20, 30 (recommended by the supplier), 40 and 60 g/L with stirring in the heating magnetic plate at 45 °C until completely dissolved. Then, the enzymatic pretreatment was optimized by studying different transglutaminase concentrations and incubation time at 40 °C. In particular, the enzymatic pretreatment was studied adding transglutaminase concentrations of 2, 3, 5, 7 and 10 g/L, and incubation times at 40 °C (using an agitated heating water bath covered with aluminium foil to avoid light during enzymatic conversion) after 20, 40, 60 and 80 min. After each incubation, the pre-treated gelatin was heated at 80 °C using the heating magnetic plate.

All the next processing steps were conducted inside a laminar flow cabinet (previously disinfected with 70% ethanol and exposed to UV light for 30 min) to avoid contamination of the prepared substrates. Substrates were allowed to cool down (after thermal treatments) to 45 °C. Afterwards, they were distributed into polypropylene trays (120 × 173 × 38 mm, 750 mL capacity; Solplast, Lorca, Spain) with ≈150 mL per tray and then allowed to cool down at room temperature to allow gelation. To select the appropriate substrate, the physical and sensory properties of each substrate were evaluated after 24 h.

#### 3.2.2. Optimization and Selection of the Packaging System

Different packaging systems were studied to ensure a hygienic design that avoids contamination of the substrates and microgreens during germination. Packaging system 1 (Figure 1(1)) consisted of the sown tray covered with another empty tray placed upside down, and the edges of both trays were sealed discontinuously with adhesive tape to allow some gas exchange. Packaging system 2 (Figure 1(2)) was the same as system 1, except that the adhesive tape sealed the edges of the trays continuously, and a 5-mm hole was made in the center of the upper tray (to further increase gas exchange) using a sharp punch. Packaging system 3 (Figure 1(3)) was the same as system 2, but a 0.22-µm syringe filter (CHROMAFIL^®^ Xtra PA-20/25, 0.2 µm) was inserted into the 5-mm hole, allowing gas exchange while ensuring that the incoming air was free of environmental microorganisms. Packaging system 4 (Figure 1(4)) was the same as system 2, but the entire system was additionally packaged using a PLA flow-pack (35 µm thick; CO_2_ and O_2_ permeabilities of 2170 and 400 cm^3^/m^2^·day·atm, respectively; Plásticos Romero, Murcia, Spain).

### 3.3. Effect of Supplementation of the Edible Substrate with EOs During Microgreen Production

The EOs were selected from a collection of nearly 100 EOs available in our research group, based on preliminary informal sensory evaluations by our Group. The three possible combinations selected were: EOs1, lemon:grapefruit:lemongrass:mint 4:3:3:1 (*volume* (*v*):*v*:*v*:*v*); EOs2, cinnamon:orange:ginger 1:4:3 (*v*:*v*:*v*); EOs3, cinnamon:orange:oregan 1:5:3 (*v*:*v*:*v*). Informal sensory tests were performed to select the most appropriate and its dose. For this, a total EO concentration of 0.01% in the edible selected substrate (gellan gum at 20 g/L) was used, as these compounds are highly bioactive and can exert modulatory effects even at minimal doses. The EOs concentration was also selected based on preliminary informal sensory tests, providing a balanced flavour of the edible substrate. The EOs3 (hereinafter referred to as EOS) was chosen due to its compatibility with mustard flavour and the technological functionalities (antimicrobial, antioxidant, etc.) of these EOs. In particular, oregano EO is rich in compounds such as thymol and carvacrol, which exhibit strong antioxidant, antibacterial, and antifungal activity [52,53,54]. Orange EO generally shows lower antibacterial and antioxidant activity compared with oregano EO; however, orange oil possesses some antioxidant properties that may be of interest for our product [52,55]. Cinnamon EO contains bioactive compounds such as cinnamaldehyde and eugenol, which have been widely reported to display potent antimicrobial and antioxidant activities. These properties may contribute to enhancing both the safety and oxidative stability of the final formulation, supporting its inclusion in the selected combination.

To reduce contamination risk, the selected EOs mix was filtered with a sterile 0.22 µm membrane filter. Sterile Tween 80 was added to the EOs mix at 50 g/L to facilitate the subsequent EO dispersion in the edible substrate. The EOs:Tween 80 mix was then added to the liquid edible substrate (once it reached 45 °C during cooling after the thermal treatments, as previously explained) and mixed in the magnetic plate until completely dissolved. Then, the EO-supplemented substrate was poured into the trays, followed by the remaining steps (seed sowing, etc.) as previously described. Red mustard was selected for this EOs-supplementation experiment since it was more interesting from its highest bioactive compound contents (see Section 2.2.3).

During microgreens growth (conditions detailed in Section 2.1), several determinations were made (as described in the following sections) after 3, 6 and 9 days. In addition, samples were taken at those sampling times, frozen with liquid nitrogen and stored at −80 °C until further analyses of pigments and bioactive compounds.

### 3.4. Biometric Parameters: Germination, Hypocotyl Length, Cotyledon Surface Area, Fresh Weight, Moisture Content and Pigments

Germination was assessed 48 h and 72 h after sowing. Seeds were considered germinated when a visible radicle of at least 2 mm was observed.

Hypocotyl length was measured from photographs of the microgreens using ImageJ software (v1.54p, Wayne Rasband; Bethesda, USA). Measurements were performed on 30 replicates (1 replicate (*n*) = 1 microgreen) per treatment. Cotyledon surface area was also determined from the same photographic images using ImageJ software. A total of 30 replicates (1 replicate (*n*) = 1 microgreen) were analyzed for each treatment.

Fresh weight of each seedling was determined using a precision balance (±0.0001 g; FV-220, Gram Precision SL, Barcelona, Spain) on 30 replicates per treatment (1 replicate (*n*) = 1 microgreen). Dry matter (%) was measured on three replicates per treatment (*n* = 3), each consisting of approximately 30 microgreens per replicate, after drying at 105 °C until constant weight.

For pigments (chlorophylls and carotenoids), 50 mg of frozen tissue was extracted in 1.5 mL methanol using an ultraturrax (Ultraturrax T25 basic, IKA; Berlin, Germany) at 14,000 rpm for 10 s, and incubated overnight at 4 °C. The extracts were then centrifuged (16,000× *g*, 5 min, 4 °C) and the supernatants were used as pigment extracts (the same extracts were also used for the total phenolic content, total flavonoid content and total antioxidant capacity). Finally, absorbances of extracts were measured at 470, 666 and 653 nm in a cuvette spectrophotometer. Chlorophylls (a and b) and total carotenoids (carotenes and xanthophylls) were calculated using Equations (1)–(3) [56]. The pigment concentrations obtained from these equations (expressed in µg/mL) were subsequently converted to mg/kg on a fresh weight (FW) basis.

C_a_ = (15.65 × A_665_) − (7.34 × A_652_) (1)

C_b_ = (27.05 × A_652_) − (11.21 × A_665_) (2)

Carotenoids = ((1000 × A_470_) − (2.86 × C_a_) − (129.2 × C_b_))/221 (3)

### 3.5. Total Flavonoid Content, Total Phenolic Content, Total Antioxidant Capacity and Malondialdehyde Determination

The total flavonoid content of the extract described in the previous section was determined as previously reported [57], with slight modifications. Briefly, 30 μL of extract was placed in a well of a 96-well plate (Greiner-Bio-one; Frickenhaus, Germany), mixed with 80 μL of 150 mM AlCl_3_ and allowed to react in the dark for 1 h. Finally, the absorbance was measured at 415 nm with a microplate reader (Infinite M200; Tecan, Männedorf, Switzerland). Quantification was performed using a commercial rutin standard (Sigma, St. Louis, MO, USA) and expressed as equivalents of Trolox in mg/kg on an FW basis.

The total phenolic content of the extract described in the previous section was determined as previously reported [58], with slight modifications. For the phenolic analysis, 19 μL of diluted extract was placed in a well of a 96-well plate and 29 μL of 1 N Folin–Ciocalteu was added and allowed to react for 3 min in darkness. Then, 192 μL of saline buffer (0.1 M NaOH and 0.19 M Na_2_CO_3_ in ultrafiltered water) was added and allowed to react in the dark for 2 h. Finally, the absorbance was measured at 750 nm with the microplate reader. Quantification was performed using a commercial gallic acid standard (Sigma, St. Louis, MO, USA) and expressed as equivalents of gallic acid in mg/kg on an FW basis.

The total antioxidant capacity of the extract described in the previous section was determined as previously reported [59], with slight modifications. Briefly, 21 μL of extract was placed in a well of a 96-well plate and 194 μL of pre-adjusted (to 1.10 ± 0.02 absorbance at 517 nm) DPPH (2,2-diphenyl-1-picrylhydrazyl) methanolic solution and allowed to react in the dark for 20 min. Finally, the absorbance was measured at 517 nm with the microplate reader. Quantification was performed using a commercial Trolox (6-hydroxy-2,5,7,8-tetramethylchroman-2-carboxylic acid) standard (Sigma, St. Louis, MO, USA) and expressed as equivalents of Trolox in mg/kg on an FW basis.

Malondialdehyde (MDA) content, a product of lipid peroxidation, was estimated using the thiobarbituric acid (TBA) method, which is used to determine cell membrane damage [60]. The expression of the results was milligrams of MDA per gram of fresh weight (mg MDA g^−1^ FW). The determinations were made in triplicate.

### 3.6. Phytomelatonin Content

The samples for the phytomelatonin analysis were prepared according to our methodology [61,62]. In brief, 4 mL of ethyl acetate was added to 0.2 g of dry material from the different acorn samples in PVPP tubes. Each sample was repeated three times. After overnight (15 h) in a rotating agitator at 20 °C in darkness, the samples were centrifuged for 10 min at 7500 rpm using a Sorvall RC 5B Plus centrifuge (DuPont, Wilmington, DE, USA) and then decanted into a new tube. Next, the solvent was evaporated to dryness under vacuum using a SpeedVac (ThermoSavant SPD111V, Thermo-Fisher Sci, Waltham, MA, USA). The final residue was resuspended in 1 mL of acetonitrile using an agitator vortex to ensure homogeneity before being filtered (PTFE filter 0.22 μm). Finally, the phytomelatonin content of the samples was analyzed by LC-FLUO.

Phytomelatonin was quantified using a high-performance liquid chromatograph (HPLC). An online degasser, quaternary pump, autosampler, thermo-stated column, and a Phenomenex-Luna ODS2 S5 (150 × 4.6 mm) column were all included in the Jasco model 2000 HPLC (Jasco Co., Tokyo, Japan). The instrument was connected to a Jasco FP-2020-Plus fluorescence detector (λ_excitation_ = 280 nm, λ_emission_ = 350 nm). The isocratic mobile phase consisted of acidified water (85%) and methanol (15%), at a flow rate of 0.5 mL/min and a column temperature of 36 °C. The retention times compared with standard melatonin (Rt = 11.2 min) and an in-line fluorescence analysis of the molecule’s excitation and emission spectra using the Jasco ChromNav 2.0 Spectra Manager software were applied to identify melatonin in our samples. Additionally, an Agilent LC chromatograph (Agilent Technologies, Santa Clara, CA, USA) coupled to a 6550 Q-TOF mass spectrometer (LC/QTOF-MS) was used to confirm the identity of melatonin.

### 3.7. Statistical Analyses

The results obtained were analyzed with an ANOVA (SPSS v.31.0.1.0; New York, NY, USA). Statistical significances were considered at *p* of 0.05, and means were separated from Tukey’s multiple range test.

## 4. Conclusions

This study demonstrates that an edible gellan gum substrate enables effective production of mustard microgreens within an edible-substrate-packaging system based on the “*farm on the fork*” concept. Gellan gum (20 g/L) supported germination and growth without irrigation and withstood sterilization. Clear cultivar-dependent differences were observed: white mustard produced the greatest biomass and phenolic content, while red mustard showed the highest flavonoids, carotenoids, antioxidant capacity, phytomelatonin and malonaldehyde, highlighting its superior nutraceutical profile. Green mustard displayed intermediate characteristics. The edible substrate allowed normal pigment and antioxidant accumulation, confirming that functional quality is maintained—or even enhanced—compared with standard substrates. Essential oil supplementation caused no negative effects on growth and only minor changes in dry matter, with slight transient increases in antioxidant metabolites, suggesting a mild elicitation effect. Overall, the gellan gum–packaging system offers a sustainable, ready-to-eat microgreen production approach. Further research should focus on optimizing controlled EO release, evaluating shelf life and sensory quality, and validating the system across additional species and production scales.

## Figures and Tables

**Table 1:** Fresh weight (mg, *n* = 30), hypocotyl length (cm, *n* = 30), cotyledon surface area (mm^2^, *n* = 30) and dry weight (%, *n* = 3) of different mustard types during microgreen growth (22 °C, 16:8 h light:dark photoperiod) (mean ± SD). Different capital letters indicate differences (*p* < 0.05) among mustard types at the same growth time. Different lowercase letters indicate differences (*p* < 0.05) among the different growth times for the same mustard type.

	Day 3	Day 6	Day 9 (*microgreen*)
**Fresh weight**			
Red mustard	11.39 ± 3.75 ^Ba^	17.90 ± 5.91 ^Ba^	17.91 ± 4.74 ^Ca^
Green mustard	14.39 ± 4.10 ^Bb^	23.70 ± 6.36 ^Bab^	31.51 ± 5.29 ^Ba^
White mustard	28.45 ± 8.62 ^Ab^	66.01 ± 9.59 ^Aa^	63.13 ± 7.54 ^Aa^
**Hypocotyl length**			
Red mustard	0.68 ± 0.14 ^Ab^	1.68 ± 0.41 ^Aa^	1.70 ± 0.33 ^Ca^
Green mustard	0.83 ± 0.20 ^Ac^	2.17 ± 0.35 ^Ab^	2.93 ± 0.37 ^Ba^
White mustard	1.04 ± 0.34 ^Ac^	2.62 ± 0.46 ^Ab^	3.52 ± 0.63 ^Aa^
**Cotyledon surface area**			
Red mustard	6.22 ± 1.79 ^Ba^	9.02 ± 2.95 ^Ca^	11.01 ± 3.76 ^Ca^
Green mustard	11.74 ± 3.43 ^Bb^	18.49 ± 4.52 ^Bab^	20.44 ± 3.63 ^Ba^
White mustard	20.21 ± 3.80 ^Ab^	36.64 ± 5.84 ^Aa^	43.48 ± 7.48 ^Aa^
**Dry weight**			
Red mustard	10.44 ± 2.28 ^Ba^	7.73 ± 1.50 ^Aab^	3.54 ± 0.89 ^Bb^
Green mustard	16.83 ± 6.15 ^Aa^	7.38 ± 2.14 ^Ab^	6.75 ± 0.54 ^ABb^
White mustard	17.00 ± 2.70 ^Aa^	10.88 ± 4.74 ^Ab^	9.23 ± 1.04 ^Ab^

**Table 2:** Chlorophyll a (mg/kg FW), chlorophyll b (mg/kg FW) and total carotenoid contents (mg/kg FW) of different mustard types during microgreen growth (22 °C, 16:8 h light:dark photoperiod) (mean (*n* = 3) ± SD). Different capital letters indicate differences (*p* < 0.05) among mustard types at the same growth time. Different lowercase letters indicate differences (*p* < 0.05) among the different growth times for the same mustard type.

	Day 3	Day 6	Day 9 (*microgreen*)
**Chlorophyll a**			
Red mustard	122.8 ± 19.9 ^Ac^	316.7 ± 34.2 ^Ab^	401.1 ± 27.3 ^Aa^
Green mustard	68.5 ± 8.8 ^Bb^	132.1 ± 15.4 ^Ba^	105.6 ± 34.8 ^Ca^
White mustard	115.0 ± 24.7 ^Ab^	126.1 ± 23.6 ^Bab^	172.5 ± 9.9 ^Ba^
**Chlorophyll b**			
Red mustard	46.4 ± 11.1 ^Ac^	121.1 ± 22.9 ^Ab^	164.5 ± 7.9 ^Aa^
Green mustard	23.2 ± 8.1 ^Ab^	58.7 ± 12.8 ^Ba^	55.2 ± 10.3 ^Ba^
White mustard	28.8 ± 18.7 ^Ab^	44.1 ± 13.7 ^Bab^	68.8 ± 10.9 ^Ba^
**Total carotenoids**			
Red mustard	26.0 ± 5.3 ^ABc^	56.7 ± 2.7 ^Ab^	76.4 ± 3.1 ^Aa^
Green mustard	15.8 ± 6.5 ^Ba^	19.5 ± 5.5 ^Ba^	11.6 ± 8.2 ^Ba^
White mustard	29.7 ± 8.1 ^Aa^	24.5 ± 12.6 ^Ba^	20.3 ± 8.2 ^Ba^

**Table 3:** Total phenolic content (mg/g FW), total flavonoid content (mg/g FW), total antioxidant capacity (µmol TE/g FW), malondialdehyde (nmol/g FW) and phytomelatonin contents (ng PTMEL/g FW) of different mustard types during microgreen growth (22 °C, 16:8 h light:dark photoperiod) (mean (*n* = 3) ± SD). Different capital letters indicate differences (*p* < 0.05) among mustard types at the same growth time. Different lowercase letters indicate differences (*p* < 0.05) among the different growth times for the same mustard type.

	Day 3	Day 6	Day 9 (*microgreen*)
**Total flavonoid content**			
Red mustard	4.93 ± 0.02 ^Aa^	4.41 ± 0.02 ^Ac^	4.56 ± 0.10 ^Ab^
Green mustard	3.89 ± 0.01 ^Bb^	3.91 ± 0.04 ^Bb^	4.01 ± 0.07 ^Ba^
White mustard	3.59 ± 0.03 ^Cc^	3.85 ± 0.04 ^Bb^	4.01 ± 0.01 ^Ba^
**Total phenolic content**			
Red mustard	1.26 ± 0.16 ^Ca^	0.85 ± 0.02 ^Cc^	1.06 ± 0.05 ^Bb^
Green mustard	0.70 ± 0.04 ^Ba^	0.52 ± 0.03 ^Bb^	0.59 ± 0.06 ^Cb^
White mustard	1.97 ± 0.11 ^Aa^	1.98 ± 0.11 ^Aa^	1.30 ± 0.13 ^Ab^
**Total antioxidant capacity**			
Red mustard	10.90 ± 1.22 ^Aa^	7.28 ± 0.57 ^Ac^	9.02 ± 0.17 ^Ab^
Green mustard	5.80 ± 0.64 ^Ba^	4.43 ± 0.52 ^Bb^	6.04 ± 1.18 ^Ba^
White mustard	5.84 ± 0.30 ^Ba^	4.52 ± 0.19 ^Bb^	2.35 ± 0.43 ^Cc^
**Malondialdehyde**			
Red mustard	20.19 ± 0.02 ^Ab^	22.60 ± 0.01 ^Aa^	21.06 ± 0.74 ^Aab^
Green mustard	13.31 ± 0.46 ^Ba^	10.12 ± 0.17 ^Cb^	10.07 ± 1.13 ^Cb^
White mustard	20.00 ± 1.84 ^Aa^	19.16 ± 1.43 ^Ba^	14.26 ± 0.77 ^Bb^
**Phytomelatonin**			
Red mustard	16.20 ± 3.60 ^Bb^	13.43 ± 2.41 ^Bb^	25.47 ± 6.57 ^Aa^
Green mustard	28.27 ± 5.35 ^Aa^	28.30 ± 1.76 ^Aa^	9.17 ± 1.31 ^Bb^
White mustard	24.30 ± 4.43 ^Aa^	15.18 ± 1.44 ^Bb^	13.28 ± 1.50 ^Bb^

**Table 4:** Total phenolic content (mg/g FW), total flavonoid content (mg/g FW) and total antioxidant capacity (µmol TE/g FW) of red mustard during germination (22 °C, 16:8 h light:dark photoperiod) with edible substrate without (CTRL) or with essential oils (EOs) (mean (*n* = 3) ± SD). Different capital letters indicate differences (*p* < 0.05) among EO treatment at the same growth time. Different lowercase letters indicate differences (*p* < 0.05) among the different growth times for the same EO treatment.

	Day 3	Day 6	Day 9 (*microgreen*)
**Total flavonoid content**			
CTRL	4.93 ± 0.02 ^Aa^	4.41 ± 0.02 ^Bc^	4.56 ± 0.10 ^Ab^
EOs	4.39 ± 0.05 ^Bb^	4.75 ± 0.01 ^Aa^	4.34 ± 0.05 ^Bb^
**Total phenolic content**			
CTRL	1.26 ± 0.16 ^Aa^	0.85 ± 0.02 ^Bc^	1.06 ± 0.05 ^Ab^
EOs	1.10 ± 0.02 ^Ba^	1.17 ± 0.05 ^Aa^	0.89 ± 0.02 ^Bb^
**Total antioxidant capacity**			
CTRL	10.90 ± 1.22 ^Aa^	7.28 ± 0.57 ^Ac^	9.02 ± 0.17 ^Ab^
EOs	8.35 ± 0.43 ^Ba^	8.24 ± 1.27 ^Aa^	6.32 ± 0.40 ^Ab^

## Data Availability

The original contributions presented in the study are included in the article, further inquiries can be directed to the corresponding author.

## References

[1] Waterland N.L., Moon Y., Tou J.C., Kim M.J., Pena-Yewtukhiw E.M., Park S. (2017). Mineral content differs among microgreen, baby leaf, and adult stages in three cultivars of kale. HortScience.

[2] European Union (2013). EU COMMISSION IMPLEMENTING REGULATION (EU) No 208/2013 of 11 March 2013 on traceability requirements for sprouts and seeds intended for the production of sprouts. Off. J. Eur. Union.

[3] European Union (2013). EU COMMISSION REGULATION (EU) No 210/2013 of 11 March 2013 on the approval of establishments producing sprouts pursuant to Regulation (EC) No 852/2004 of the European Parliament and of the Council. Off. J. Eur. Union.

[4] (2014). BOE Real Decreto 379/2014, de 30 de mayo, por el que se regulan las condiciones de aplicación de la normativa comunitaria en materia de autorización de establecimientos, higiene y trazabilidad, en el sector de los brotes y de las semillas destinadas a la prod. Boletín Of. Estado.

[5] FDA (2023). Guidance for Industry: Standards for the Growing, Harvesting, Packing, and Holding of Sprouts for Human Consumption.

[6] MAPA (2016). Guía de Buenas Prácticas de Higiene en la Producción Primaria de Brotes Vegetales.

[7] Artés–Hernández F., Miranda-Molina F.D., Klug T.V., Martínez–Hernández G.B. (2022). Enrichment of glucosinolate and carotenoid contents of mustard sprouts by using green elicitors during germination. J. Food Compos. Anal..

[8] Alloggia F.P., Bafumo R.F., Ramirez D.A., Maza M.A., Camargo A.B. (2023). Brassicaceae microgreens: A novel and promissory source of sustainable bioactive compounds. Curr. Res. Food Sci..

[9] Manchester L.C., Tan D.-X., Reiter R.J., Park W., Monis K., Qi W. (2000). High levels of melatonin in the seeds of edible plants: Possible function in germ tissue protection. Life Sci..

[10] Bafumo R.F., Alloggia F.P., Ramirez D.A., Maza M.A., Camargo A.B., Ramirez D.A., Camargo A.B., Maza M.A., Fontana A., Moreno D.A. (2024). Optimal *Brassicaceae* family microgreens from a phytochemical and sensory perspective. Food Res. Int..

[11] Topalcengiz Z., Chandran S., Gibson K.E. (2024). A comprehensive examination of microbial hazards and risks during indoor soilless leafy green production. Int. J. Food Microbiol..

[12] Balik S., Dasgan H.Y., Ikiz B., Gruda N.S. (2024). The performance of growing-media-shaped microgreens: The growth, yield, and nutrient profiles of broccoli, red beet, and black radish. Horticulturae.

[13] Di Gioia F., Renna M., Santamaria P. (2017). Sprouts, Microgreens and “baby leaf” vegetables. Food Engineering Series.

[14] Paradiso V.M., Castellino M., Renna M., Gattullo C.E., Calasso M., Terzano R., Allegretta I., Leoni B., Caponio F., Santamaria P. (2018). Nutritional characterization and shelf-life of packaged microgreens. Food Funct..

[15] Cebrián-Lloret V., Martínez-Abad A., López-Rubio A., Martínez-Sanz M. (2024). Exploring alternative red seaweed species for the production of agar-based hydrogels for food applications. Food Hydrocoll..

[16] Calvarro J., Perez-Palacios T., Ruiz J. (2016). Modification of gelatin functionality for culinary applications by using transglutaminase. Int. J. Gastron. Food Sci..

[17] Gomes D., Batista-Silva J.P., Sousa A., Passarinha L.A. (2023). Progress and opportunities in gellan gum-based materials: A review of preparation, characterization and emerging applications. Carbohydr. Polym..

[18] Baenas N., García-Viguera C., Moreno D.A. (2014). Biotic elicitors effectively increase the glucosinolates content in *Brassicaceae* sprouts. J. Agric. Food Chem..

[19] Hassini I., Baenas N., Moreno D.A., Carvajal M., Boughanmi N., Martinez Ballesta M.D.C. (2017). Effects of seed priming, salinity and methyl jasmonate treatment on bioactive composition of *Brassica oleracea* var. capitata (white and red varieties) sprouts. J. Sci. Food Agric..

[20] Mutlu-Ingok A., Devecioglu D., Dikmetas D.N., Karbancioglu-Guler F., Capanoglu E. (2020). Antibacterial, antifungal, antimycotoxigenic, and antioxidant activities of essential oils: An updated review. Molecules.

[21] Viacava G.E., Roura S.I. (2015). Principal component and hierarchical cluster analysis to select natural elicitors for enhancing phytochemical content and antioxidant activity of lettuce sprouts. Sci. Hortic..

[22] Evensen E., Teng Z., Mao Y., Chen P.Y., Ortiz I., Li Y., Yang T., Fonseca J.M., Wang Q., Luo Y. (2025). Optimizing microgreen cultivation through post-crosslinked alginate-gellan gum hydrogel substrates with enhanced porosity and structural integrity. Int. J. Biol. Macromol..

[23] Gvozdenac S., Indjic D., Vukovic S. (2013). Phytotoxicity of chlorpyrifos to white mustard (*Sinapis alba* L.) and maize (*Zea mays* L.): Potential indicators of insecticide presence in water. Pestic. Fitomedicina.

[24] Khaliq G., Saleh A., Bugti G.A., Hakeem K.R. (2020). Guggul gum incorporated with basil essential oil improves quality and modulates cell wall-degrading enzymes of jamun fruit during storage. Sci. Hortic..

[25] Pavan B., Naik K., Sekhar G., Shiva G., Rajulu G., Akhila L., Deepika S., Suryakumari A., Harshini K. (2022). Effect of growth and yield of mustard (*Brassica juncea*) microgreens on different growing media in indoor conditions. Int. J. Res. Agron..

[26] Sánchez-Pérez M.I., Yazmín Muñoz-Mejía C., Quiroz-Velásquez J.C., Mayek-Pérez N., Hernández-Mendoza J.L. (2010). Physical-chemical changes during maize seed germination. Rev. Mex. Ciencias Agrícolas.

[27] Hanway J.J., Thompson H.E. (1967). How a Soybean Plant Develops.

[28] Brazaitytė A., Miliauskienė J., Vaštakaitė-Kairienė V., Sutulienė R., Laužikė K., Duchovskis P., Małek S. (2021). Effect of different ratios of blue and red led light on brassicaceae microgreens under a controlled environment. Plants.

[29] Kyriacou M.C., Rouphael Y., Di Gioia F., Kyratzis A., Serio F., Renna M., De Pascale S., Santamaria P. (2016). Micro-scale vegetable production and the rise of microgreens. Trends Food Sci. Technol..

[30] Xiao Z., Lester G.E., Luo Y., Wang Q. (2012). Assessment of vitamin and carotenoid concentrations of emerging food products: Edible microgreens. J. Agric. Food Chem..

[31] Dhaka A.S., Dikshit H.K., Mishra G.P., Tontang M.T., Meena N.L., Kumar R.R., Ramesh S.V., Narwal S., Aski M., Thimmegowda V. (2023). Evaluation of growth conditions, antioxidant potential, and sensory attributes of six diverse microgreens species. Agriculture.

[32] Alloggia F.P., Bafumo R.F., Ramírez D.A., Heredia Martín J.P., Maza M.A., Camargo A.B. (2025). Enhancement of yield and functional quality of *Brassica* microgreens: Effects of fertilization and substrate. Food Chem..

[33] Baenas N., Gómez-Jodar I., Moreno D.A., García-Viguera C., Periago P.M. (2017). Broccoli and radish sprouts are safe and rich in bioactive phytochemicals. Postharvest Biol. Technol..

[34] Ben Saad R., Ben Romdhane W., Wiszniewska A., Baazaoui N., Taieb Bouteraa M., Chouaibi Y., Alfaifi M.Y., Kačániová M., Čmiková N., Ben Hsouna A. (2024). *Rosmarinus officinalis* L. essential oil enhances salt stress tolerance of durum wheat seedlings through ROS detoxification and stimulation of antioxidant defense. Protoplasma.

[35] Li Z., Di H., Cheng W., Zhang Y., Ren G., Ma J., Yang J., Huang Z., Tang Y., Zheng Y. (2023). Variation in health-promoting compounds and antioxidant activities in mustard (*Brassica juncea*) sprouts. Sci. Hortic..

[36] Craver J.K., Gerovac J.R., Lopez R.G., Kopsell D.A. (2017). Light intensity and light quality from sole-source light-emitting diodes impact phytochemical concentrations within *Brassica* Microgreens. J. Am. Soc. Hortic. Sci..

[37] Gudžinskaitė I., Laužikė K., Pukalskas A., Samuolienė G. (2024). The effect of light intensity during cultivation and postharvest storage on mustard and kale microgreen quality. Antioxidants.

[38] Ali V., Mandal J., Vyas D. (2024). Insights into light-driven dynamics of phytochemicals in sprouts and microgreens. Plant Growth Regul..

[39] Frosch S., Mohr H. (1980). Analysis of light-controlled accumulation of carotenoids in mustard (*Sinapis alba* L.) seedlings. Planta.

[40] Von Lintig J., Welsch R., Bonk M., Giuliano G., Batschauer A., Kleinig H. (1997). Light-dependent regulation of carotenoid biosynthesis occurs at the level of phytoene synthase expression and is mediated by phytochrome in *Sinapis alba* and *Arabidopsis thaliana* seedlings. Plant J..

[41] Welsch R., Beyer P., Hugueney P., Kleinig H., von Lintig J. (2000). Regulation and activation of phytoene synthase, a key enzyme in carotenoid biosynthesis, during photomorphogenesis. Planta.

[42] Johnson J.B., Mani J.S., Broszczak D., Prasad S.S., Ekanayake C.P., Strappe P., Valeris P., Naiker M. (2021). Hitting the sweet spot: A systematic review of the bioactivity and health benefits of phenolic glycosides from medicinally used plants. Phyther. Res..

[43] Lee S., Lee J., Lee H., Sung J. (2019). Relative protective activities of quercetin, quercetin-3-glucoside, and rutin in alcohol-induced liver injury. J. Food Biochem..

[44] Park C.H., Park Y.E., Yeo H.J., Kim J.K., Park S.U. (2020). Effects of light-emitting diodes on the accumulation of phenolic compounds and glucosinolates in *Brassica juncea* sprouts. Horticulturae.

[45] Aguilera Y., Herrera T., Benítez V., Arribas S.M., López De Pablo A.L., Esteban R.M., Martín-Cabrejas M.A. (2015). Estimation of scavenging capacity of melatonin and other antioxidants: Contribution and evaluation in germinated seeds. Food Chem..

[46] Saleh H.M., Hassan A.A., Mansour E.H., Fahmy H.A., El-Bedawey A.E.F.A. (2019). Melatonin, phenolics content and antioxidant activity of germinated selected legumes and their fractions. J. Saudi Soc. Agric. Sci..

[47] Aguilera Y., Herrera T., Liébana R., Rebollo-Hernanz M., Sanchez-Puelles C., Martín-Cabrejas M.A. (2015). Impact of melatonin enrichment during germination of legumes on bioactive compounds and antioxidant activity. J. Agric. Food Chem..

[48] Arnao M.B., Hernández-Ruiz J. (2018). Phytomelatonin, natural melatonin from plants as a novel dietary supplement: Sources, activities and world market. J. Funct. Foods.

[49] Stege P.W., Sombra L.L., Messina G., Martinez L.D., Silva M.F. (2010). Determination of melatonin in wine and plant extracts by capillary electrochromatography with immobilized carboxylic multi-walled carbon nanotubes as stationary phase. Electrophoresis.

[50] Moura R.C., Amaral B.D., Lima N.K., Lopes A.D., Carmo D.F., Guesdon I.R., Bardales-Lozano R.M., Schwartz G., Dionisio L.F., Ávila M.D. (2025). Phytotoxicity of *Piper marginatum* Jacq. essential oil on detached leaves and post-emergence of plants. Rev. Bras. Eng. Agrícola Ambient..

[51] Singh P., Singh S., Kapoor I.P.S., Singh G., Isidorov V., Szczepaniak L. (2013). Chemical constitution and allelopathic effects of *Curcuma zedoaria* essential oil on lettuce achenes and tomato seeds. Food Biosci..

[52] Radünz M., Mota Camargo T., dos Santos Hackbart H.C., Inchauspe Correa Alves P., Radünz A.L., Avila Gandra E., da Rosa Zavareze E. (2021). Chemical composition and in vitro antioxidant and antihyperglycemic activities of clove, thyme, oregano, and sweet orange essential oils. LWT.

[53] Reyes-Jurado F., Cervantes-Rincón T., Bach H., López-Malo A., Palou E. (2019). Antimicrobial activity of Mexican oregano (*Lippia berlandieri*), thyme (*Thymus vulgaris*), and mustard (*Brassica nigra*) essential oils in gaseous phase. Ind. Crops Prod..

[54] Wu T.L., Zhang B.Q., Luo X.F., Li A.P., Zhang S.Y., An J.X., Zhang Z.J., Liu Y.Q. (2023). Antifungal efficacy of sixty essential oils and mechanism of oregano essential oil against *Rhizoctonia solani*. Ind. Crops Prod..

[55] Alvarez M.V., Pérez-Gago M.B., Taberner V., Settier-Ramírez L., Martínez-Blay V., Palou L. (2023). Postharvest application of novel bio-based antifungal composite edible coatings to reduce sour rot and quality losses of ‘Valencia’ oranges. Coatings.

[56] Wellburn A.R. (1994). The spectral determination of chlorophylls a and b, as well as total carotenoids, using various solvents with spectrophotometers of different resolution. J. Plant Physiol..

[57] Meda A., Lamien C.E., Romito M., Millogo J., Nacoulma O.G. (2005). Determination of the total phenolic, flavonoid and proline contents in Burkina Fasan honey, as well as their radical scavenging activity. Food Chem..

[58] Singleton V.L., Rossi J.A. (1965). Colorimetry of total phenolics with phosphomolybdic-phosphotungstic acid reagents. Am. J. Enol. Vitic..

[59] Brand-Williams W., Cuvelier M.E., Berset C. (1995). Use of a free radical method to evaluate antioxidant activity. LWT Food Sci. Technol..

[60] Niehaus W.G., Samuelsson B. (1968). Formation of malonaldehyde from phospholipid arachidonate during microsomal lipid peroxidation. Eur. J. Biochem..

[61] Cano A., Hernández-Ruiz J., Arnao M.B. (2024). Common methods of extraction and determination of phytomelatonin in plants. Methods Mol. Biol..

[62] Arnao M.B., Hernández-Ruiz J. (2009). Assessment of different sample processing procedures applied to the determination of melatonin in plants. Phytochem. Anal..

